# The postmarsupial development of *Porcellio siculoccidentalis*, with some data on reproductive biology (Crustacea, Isopoda, Oniscidea)

**DOI:** 10.3897/zookeys.176.2369

**Published:** 2012-03-20

**Authors:** Giuseppe Montesanto, Giuseppina Musarra Pizzo, Domenico Caruso, Bianca M. Lombardo

**Affiliations:** 1 University of Catania, Department of Biological, Geological and Environmental Sciences, I-95124 Catania, Italy

**Keywords:** Terrestrial isopods, postmarsupial mancas, fecundity, fertility, larvae

## Abstract

In the broader context of research on the Sicilian *Porcellio imbutus*-complex, the postmarsupial development of *Porcellio siculoccidentalis* Viglianisi, Lombardo & Caruso, 1992 was studied in detail. This research was conducted in the laboratory under controlled conditions, allowing us to follow the stages of development, from the formation of the marsupium in ovigerous females until the larval stages and development of the seventh pair of legs. The timing of developmental stages and the morphological modifications of appendages in the postmarsupial manca stages (M I–M III) are described. The manca stage M I had a duration of about one hour. Ovigerous females were collected and reared separately, and the number of parturial molts in the absence of males was counted. The results showed a maximum of four successive parturial molts. Fecundity and fertility were evaluated as the number of eggs and embryos, respectively, inside the marsupium of the ovigerous females. Both parameters were positively correlated with the size of the females. The maximum numbers of eggs and embryos in the marsupium were 113 and 141, respectively. Data describing the total number of postmarsupial mancas released per month indicated that the highest release occurred in April.

## Introduction

*Porcellio siculoccidentalis* Viglianisi, Lombardo & Caruso, 1992 is a species of terrestrial isopod, belonging to the *Porcellio imbutus*-complex, whose biology is still largely unknown. It was recently described in a study (Viglianisi et al. 1992) verifying the validity of *Porcellio imbutus* Budde-Lund, 1885, a Sicilian species that is present throughout the island. This report noted that *Porcellio imbutus* constituted four distinguishable population groups with slight but consistent morphological differences. Confirmation of these distinct groups by allozyme analysis made it necessary to consider the four groups as different species: *Porcellio imbutus*, *Porcellio siculoccidentalis*, *Porcellio baidensis* Viglianisi, Lombardo & Caruso, 1992, and *Porcellio hyblaeus* Viglianisi, Lombardo & Caruso, 1992. *Porcellio siculoccidentalis* can be quite easily separated from the other species by examination of the male secondary sexual characters (see Viglianisi et al. 1992). Specifically, its pleopod 1 exopodite has a rectangular internal lobe with an obliquely truncated apex, and the pereopod 7 has a rounded bump in the carpus.

During studies of the isopod fauna of Sicily, we found two large populations of *Porcellio siculoccidentalis* from two different holm-oak woods (*Quercus ilex*), one on the Mount San Giuliano, and one on the Mount Inici, both in Trapani province (western Sicily). The population from Mount San Giuliano was found to be infected with long Mermithid nematodes that lived in the haemocoel of the isopods; some specimens were also infected with Iridovirus. To determine whether and how these symbionts modified the biological parameters of the species, we began to study the reproductive biology of the uninfected specimens of the populations found on Mount Inici and Mount San Giuliano, so as to make future comparisons with the infected specimens from Mount San Giuliano.

The aim of this report is to clarify some biological aspects of the species. In particular, we investigated the first stages of postmarsupial development and assessed the fecundity and fertility parameters. Here, we describe the postmarsupial manca stages, and the number of successive parturial molts and births after only one mating, as well as provide some data on the total number of mancas released per year.

## Materials and methods

The animals were collected by hand with the aid of entomological forceps, from the litter and under stones of the holm-oak woods of Mount San Giuliano and Mount Inici, both in Trapani province (western Sicily). Sampling sites are indicated in Table 1. In the laboratory, appropriate breeding was carried out in plastic boxes of 35 × 60 cm, with soil that had been taken from the sampling sites and sterilized. The specimens were fed with slices of potatoes and carrots, and sterilized and previously moistened plane-tree (*Platanus acerifolia*) leaves. The breeding substrate was periodically moistened with vaporized water, and kept at constant temperature of 20°C (±1°C).

Ovigerous females were collected from the sampling sites and were bred separately. Each female was kept in a Petri dish with a substrate made of chromatography paper, periodically moistened, and fed with slices of potatoes and carrots. Each time an ovigerous female was observed during the release of the mancas from the marsupium; the mancas were followed until the first ecdysis occurred. After that, the mancas were counted, separated from the female, and reared in different Petri dishes, using the same methods as already described.

Ovigerous females were observed daily in order to record the time of the manca release. The postmarsupial mancas were also checked daily in order to observe the molting process and record the time of each manca stage.

Fifty ovigerous females were dissected in order to analyze the marsupium content; eggs and embryos were counted (embryo stages: S14-S18, *sensu* Milatovic et al. 2010) to estimate the fecundity and fertility parameters, respectively indicated by the number of oocytes in the marsupium, and by the number of embryos in relation to the number of eggs. The number of eggs and embryos in the marsupium was related to the body length of the ovigerous femals, according to AlJetlawi and Nair (1994), that worked on similar Mediterranean biotopes.

Throughout postmarsupial development, at least ten individuals that were representative of each postmarsupial manca stage were fixed in 70% ethanol for the successive descriptions and measurements. The observations and the dissections were performed using a stereomicroscope (Leica EZ4D) and dissection forceps (Dumont & Fils No. 5). The manca stages were described following the standard procedures (Araujo et al. 2004, Brum and Araujo 2007, Sokolowicz et al. 2008), including morphological study of the appendages (antenna, antennula, maxilla, maxillula, maxillipede, pereopods, and pleopods). These parts were mounted on slides, and drawings were made using the optical microscope (Ernst Leitz Wetzlar) and a *camera lucida*. The measurements were taken using the stereomicroscope (Leica M205) equipped with a digital camera (DFC420), and using the dedicated software LES ver. 3.3.0. SEM photographs were taken with a ZEISS Evo LS 10.

**Table 1.** Localities, WGS84 coordinates, and altitudes of the sampling sites.

**Locality**	**WGS84 coordinates**	**Altitude (a.s.l.)**
Monte S. Giuliano, Erice (TP)	38°2'6.92"N, 12°34'54.55"E	681 m
Monte S. Giuliano, Erice (TP)	38°2'12.30"N, 12°35'33.50"E	646 m
Monte Inici, Castel. del Golfo (TP)	38°1'1.12"N, 12°51'25.35"E	1064 m

## Results

**Figure 1.**
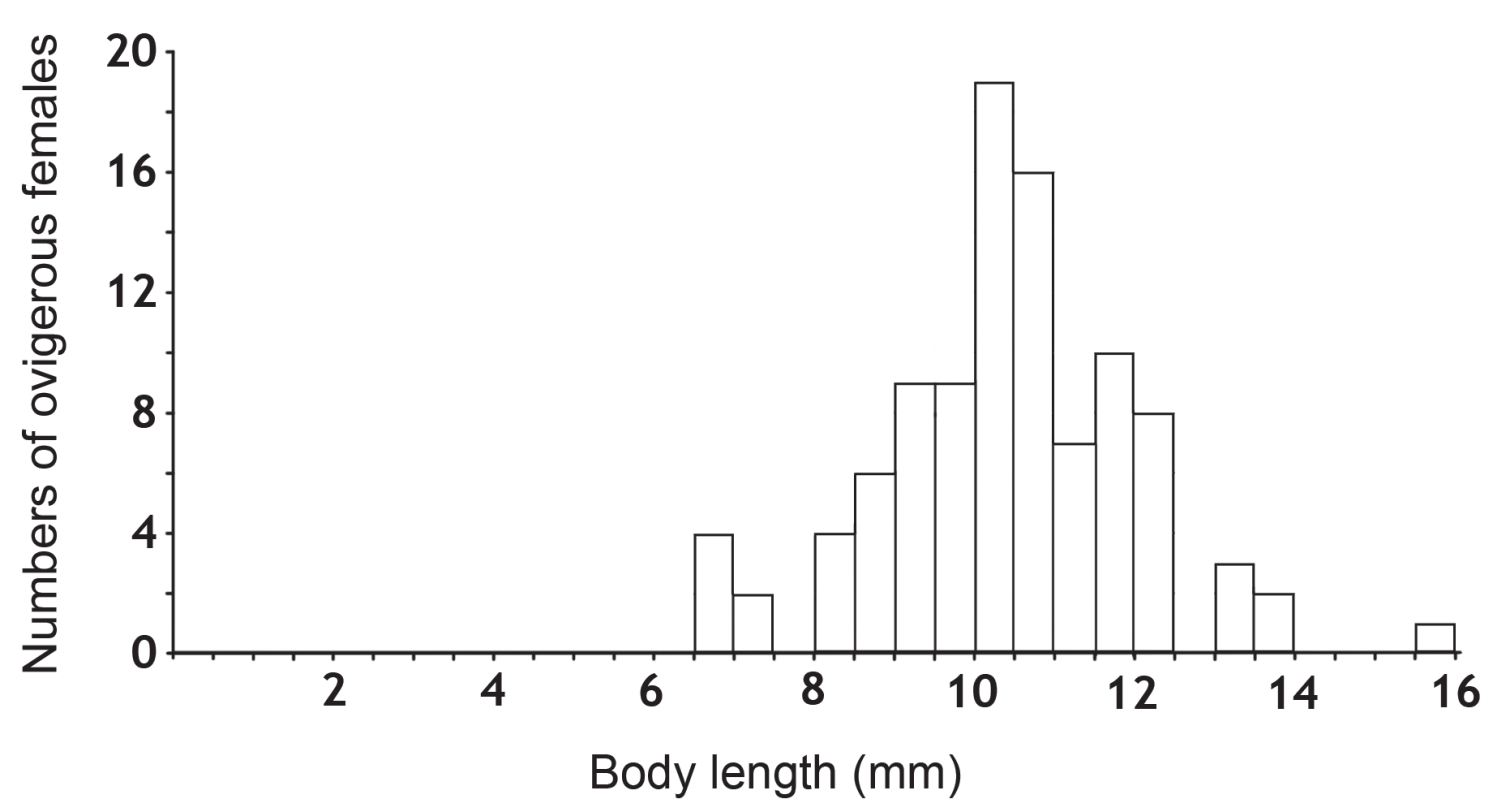
Distribution of body lengths of the ovigerous females used in the present study.

**Figure 2.**
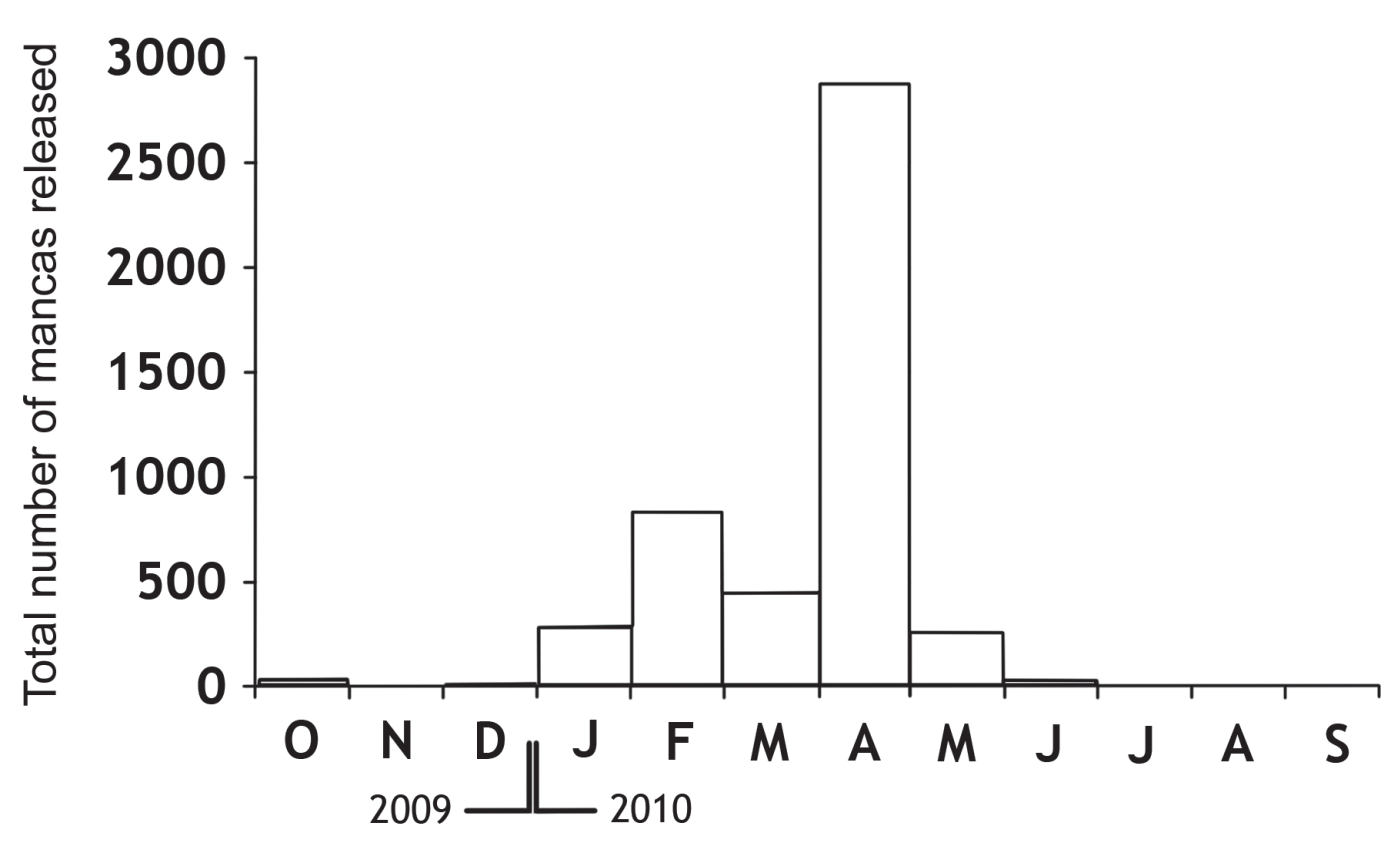
Total number of mancas released during the study period.

One hundred ovigerous females were taken in order to study their reproductive biology in the laboratory, to determine the fecundity and fertility parameters, and to describe the larval stages of the species. The body lengths of this sample ranged from 6.5 mm to 16 mm (Fig. 1). A total of 1153 specimens, including 653 females (57%) and 500 males (43%) resulted from the breeding. The births occurred in October of 2009, and between January and May of 2010, with a peak in April, during which the highest number of mancas was released (Fig. 2).

All ovigerous females showed a seasonal reproduction, with 1–4 successive parturial molts occurring before entering a non-reproductive period. The females had never successively mated with males. The times between successive parturial molts ranged from 20 to 40 days. During the reproductive period, only one parturial molt was recorded for 39 females, two successive parturial molts for 46 females, three successive parturial molts for 12 females, and four successive parturial molts were recorded for 3 females only.

The survival rate of females after the brood release was as follows: 9% of the females died before the first brood release, 72% gave only one brood release, 16% gave two successive brood releases, and only 3% gave three successive releases of mancas.

**Figure 3.**
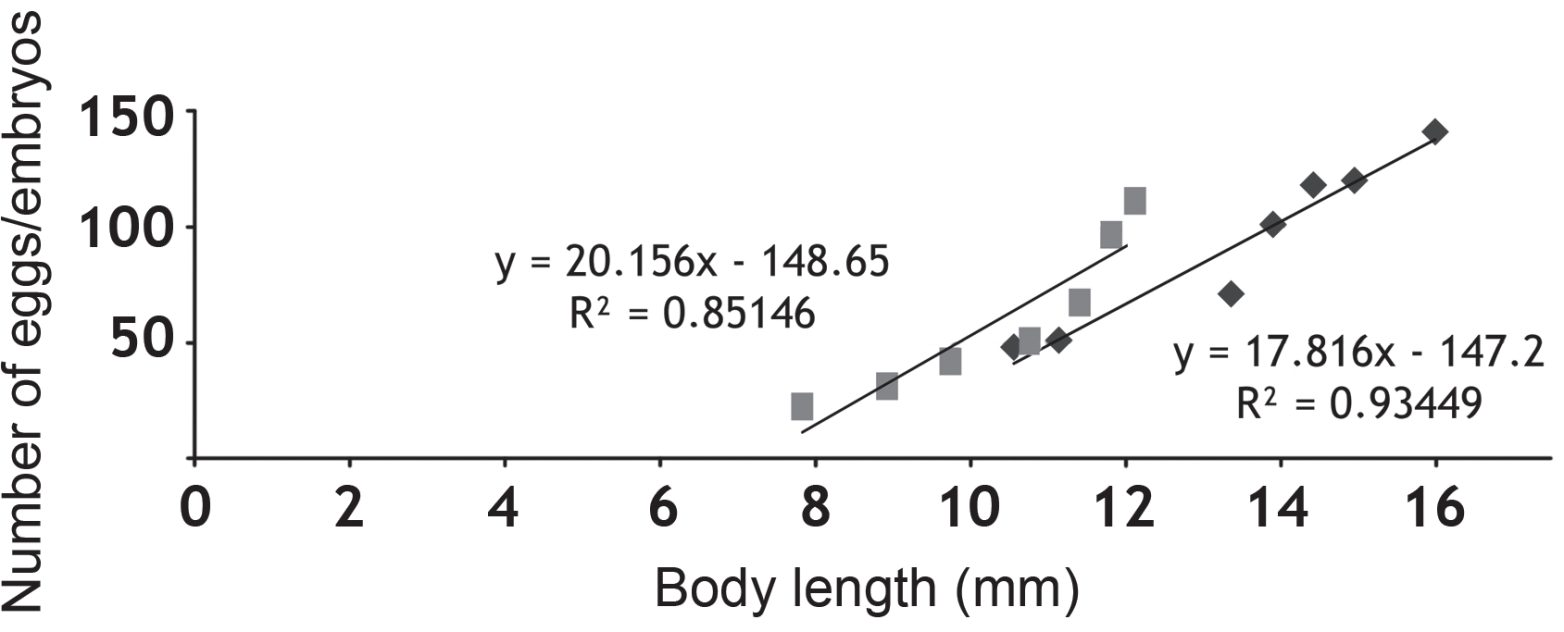
Correlation of the ovigerous females’ body lengths with the number of eggs (▒ - fecundity) and with number of embryos in the brood pouch (⬧ - fertility).

Fecundity, estimated by the number of eggs in the marsupium, showed a positive correlation with the female size (R² = 0.85), varying from 22 eggs in a female with body length of 7.9 mm, to 113 eggs in a female with body length of 12.1 mm. Fertility, estimated by the number of embryos in the marsupium, also showed a positive correlation with the size of the female (R² = 0.93), varying from 48 embryos in a female with body length of 10.56 mm, to 141 embryos in a female with a body length of 16 mm (Fig. 3).

**Figure 4.**
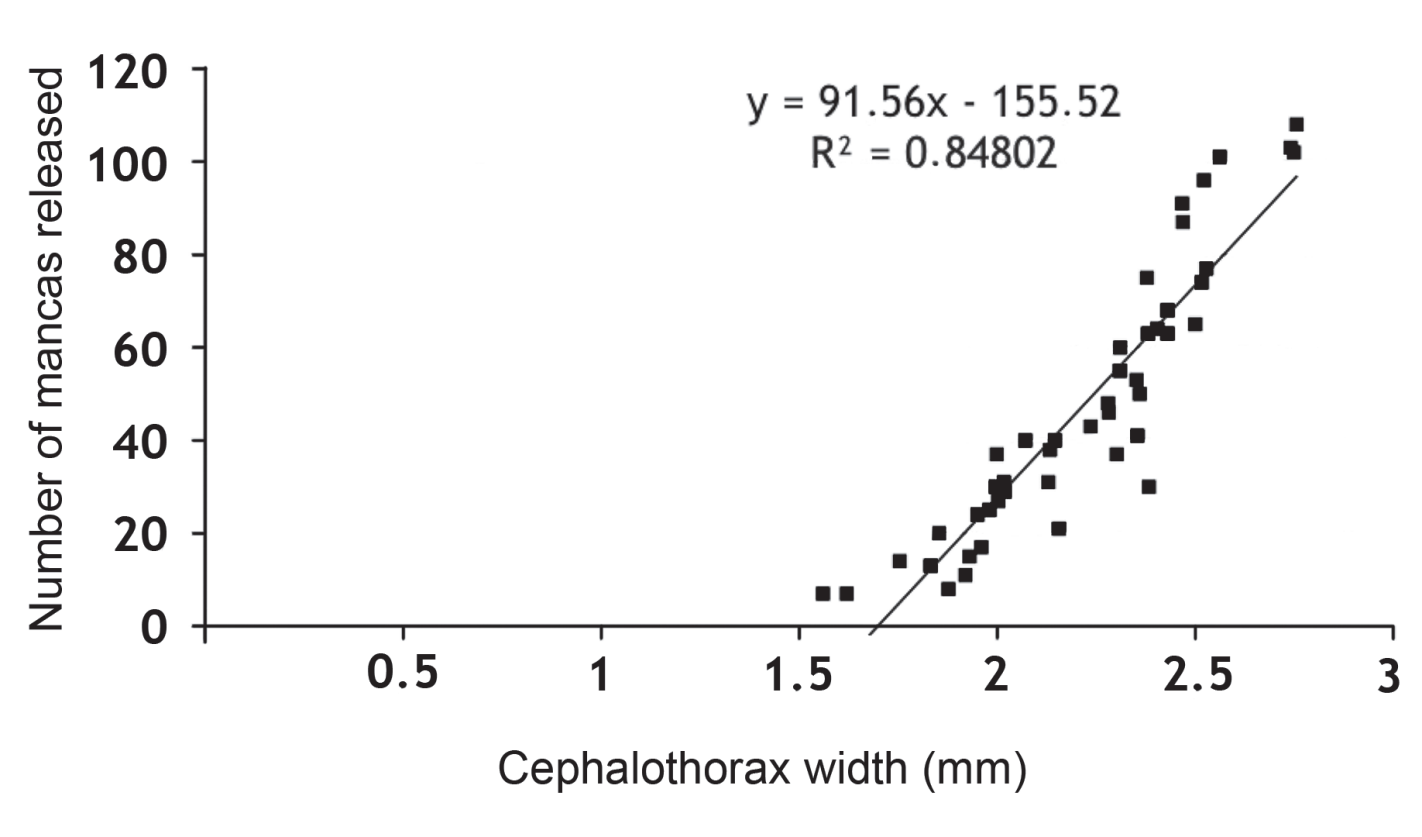
Correlation between cephalothorax widths of the ovigerous females with the number of mancas released.

A positive correlation was also observed between the number of mancas released and the cephalothorax width of the females. The number of mancas released ranged from 7 for a female with cephalothorax width of 1.62 mm, to 108 for a female with cephalothorax width of 2.76 mm, with a mean value of 48 mancas per female (Fig. 4).

*Porcellio siculoccidentalis* showed three postmarsupial larval stages, also called manca stages (M I, M II, and M III), separated by ecdysis and characterized by the absence of the first pleopods and non-functional seventh pereopods. The main characteristics that were distinctive of each stage relate to the length of the flagellum articles, the developmental level of the pereopod 7, the number of setae of pleopods 2–5, and the number of ommatidia (Table 2).

**Figure 5.**
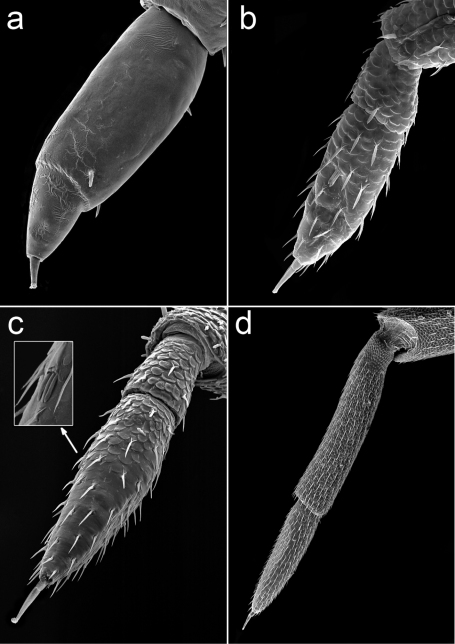
*Porcellio siculoccidentalis* Viglianisi et al., 1992. Flagellum: **a** manca stage M I (500X) **b** manca stage M II (330X) **c** manca stage M III (300X) **d** adult (65X).

**Figure 6.**
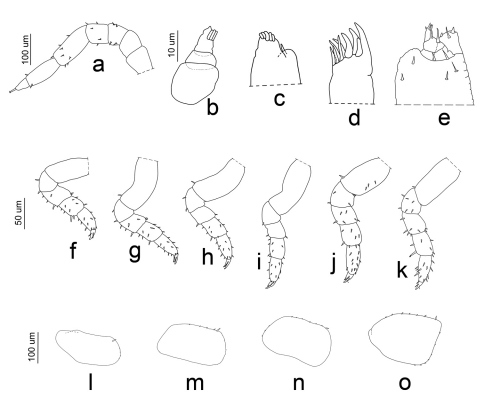
*Porcellio siculoccidentalis* Viglianisi et al., 1992. Manca stage M I: **a** antenna **b** antennula **c** maxilla **d** maxillula **e** maxillipede **f** pereopod 1 **g** pereopod 2 **h** pereopod 3 **i** pereopod 4 **j** pereopod 5 **k** pereopod 6 **l** pleopod 2 exopodite **m** pleopod 3 exopodite **n** pleopod 4 exopodite **o** pleopod 5 exopodite.

*Manca I stage (M I).* The duration ofthis stage varied from a minimum of 57 min to a maximum of 66 min, with a mean value of 62 min. The mean body length was 1.788 mm (SD: ±0.053), with a range from 1.653 mm to 1.82 mm. The mean cephalothorax width was 0.48 mm, with a minimum of 0.35 mm and a maximum of 0.53 mm. The first larval stage had no pigmentation, except for the ommatidia and little spots on the pereonite margins. Because of the transparency it was possible to observe the presence of food in the gut. Eyes had four ommatidia. Pereonite 7 and seventh pereopod were absent. The flagellum was bi-articulated and was as long as the fifth article and, in contrast to the other larval stages, the proximal article was twice as long as the apical (Figs 5a, 6a). Antennula showed three articles and three apical aesthetascs (Fig. 6b). Maxilla had one seta on the apical part, between the lateral and the medial lobes; five setae were present on the medial lobe (Fig. 6c). Maxillula had nine teeth (Fig. 6d). The maxillipede had the palp with apical setal tuft, and two setae in the basal region; the endite had two teeth and one apical seta (Fig. 6e). Pereopods 1–6 had few setae that were distributed generally on the lateral margins of the articles (Fig. 6f–k). Pleon was lacking the first pleopods. Pleopods 2–5 exopodite had one small seta on the distal margin (Fig. 6l–o).

**Figure 7.**
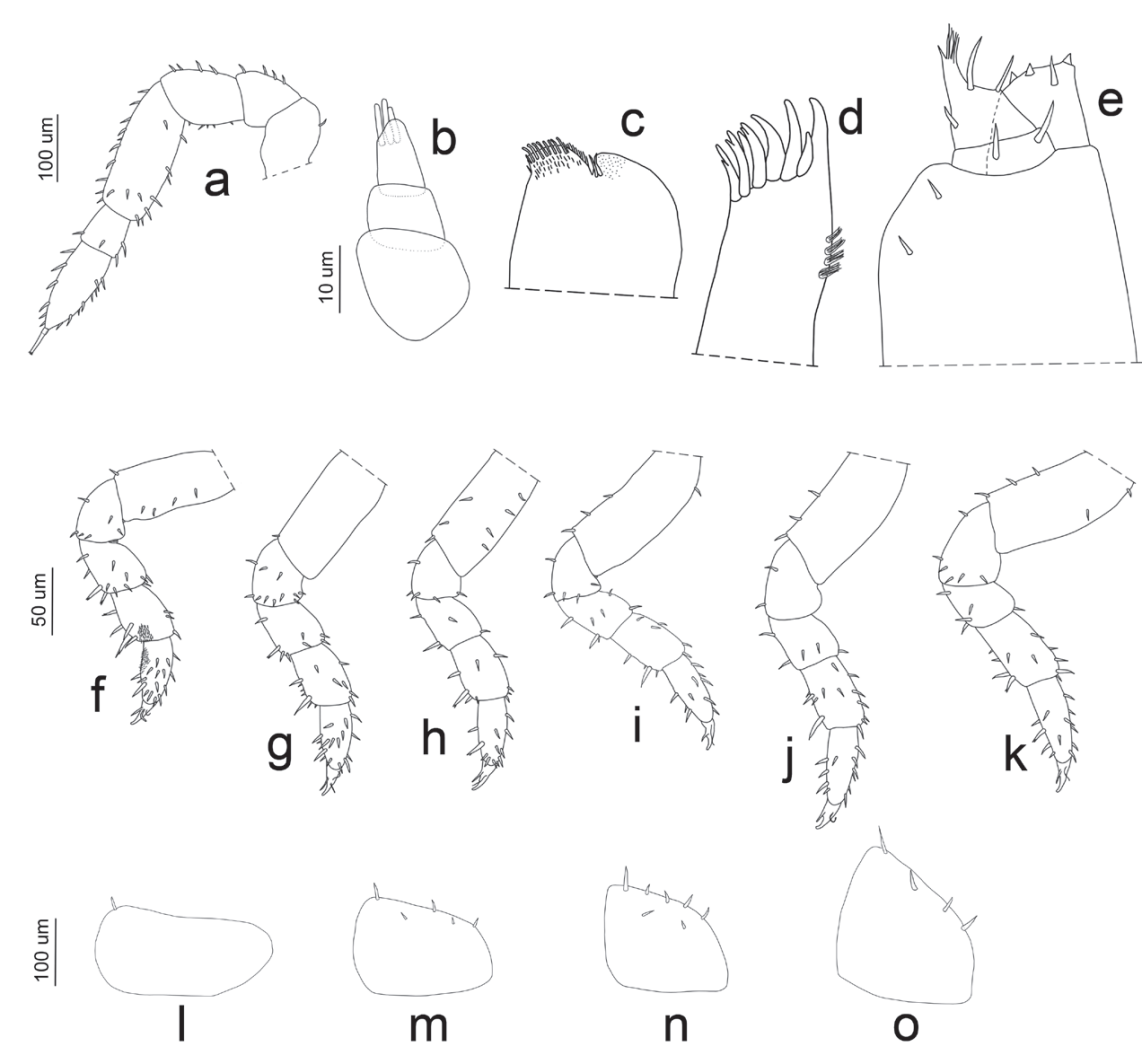
*Porcellio siculoccidentalis* Viglianisi et al., 1992. Manca stage M II: **a** antenna **b** antennula **c** maxilla **d** maxillula **e** maxillipede **f** pereopod 1 **g** pereopod 2 **h** pereopod 3 **i** pereopod 4 **j** pereopod 5 **k** pereopod 6 **l** pleopod 2 exopodite **m** pleopod 3 exopodite **n** pleopod 4 exopodite **o** pleopod 5 exopodite.

**Figure 8.**
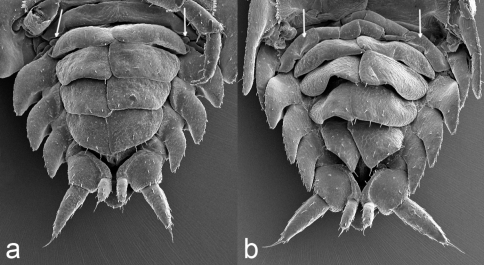
*Porcellio siculoccidentalis* Viglianisi et al., 1992. Pleon (ventral vision) and pereopods 7 (indicated with white arrows): **a** manca stage M II (80X) **b** manca stage M III (80×).

*Manca II stage (M II).* The time needed to pass from M I to M II was very short, with an average of about 62 min. The intermolt time of M II was almost 10 days, with a minimum of 8 days and a maximum of 11 days. Our M II specimens were 2.023 mm long on average (SD: ±0.098), with a minimum value of 1.63 mm and a maximum of 2.12 mm. Cephalothorax mean width was 0.62 mm, with a minimum of 0.59 mm and a maximum of 0.65 mm. Pigmentation on the pereionite margins was stronger than in M I and appeared also on the cephalothorax. Eyes had five ommatidia. Antenna article lengths showed an inversion of those of flagellum, with the distal article twice the length of the proximal (Fig. 5b, 7a). Antennula was similar to in the M I stage (Fig. 7b). Maxilla showed a high number of setae only on one lobe; the two lobes were of the same size (Fig. 7c). Maxillula had lateral setal tuft and nine teeth, three of them were bifid (Fig. 7d). The maxillipede showed palp with one apical setal tuft, and two long apical setae with one in the basal position; endite had three teeth and one apical seta (Fig. 7e). Pereonite 7 appeared. Pereopods 1–6 had stronger and more numerous setae than in the M I stage (Fig. 7f–k); pereopod 7 began to develop, but it was not possible to distinguish articles (Fig. 8a). Pleopod 1 was absent; pleopod 2 had one seta; and pleopods 3–5 had five, seven, and four setae, respectively, on the distal margin (Fig. 7l–o).

**Figure 9.**
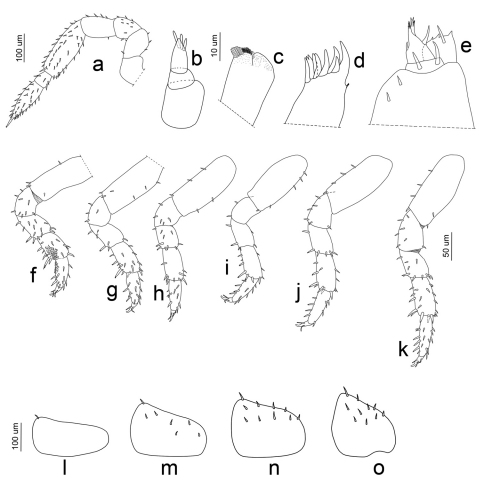
*Porcellio siculoccidentalis* Viglianisi et al., 1992. Manca stage M III: **a** antenna **b** antennula **c** maxilla **d** maxillula **e** maxillipede **f** pereopod 1 **g** pereopod 2 **h** pereopod 3 **i** pereopod 4 **j** pereopod 5 **k** pereopod 6 **l** pleopod 2 exopodite **m** pleopod 3 exopodite **n** pleopod 4 exopodite **o** pleopod 5 exopodite.

*Manca III stage (M III).* Intermolt duration of this stage was on average 11 days, with a minimum of 10 days and a maximum of 12 days. Our M III specimens were 2.465 mm long on average (SD: ±0.149), with a minimum of 2.2 mm and a maximum of 2.633 mm. Cephalothorax width varied from a minimum of 0.66 mm to a maximum of 0.796 mm, with a mean of 0.72 mm. A light brown pigmentation could be observed on the whole body dorsal surface. Eyes consisted of seven ommatidia. Antennae were similar as in the M II stage (Fig. 9a), but the distal article of the flagellum was more than twice the length of the proximal (Fig. 5c). Antennula had four aesthetascs (Fig. 9b). Maxilla had a higher number of setae on the lobe than in the M II stage (Fig. 9c). Maxillula was similar to the M II stage, but with numerous lateral setal tufts (Fig. 9d). The maxillipede was similar to the M II stage (Fig. 9e). Pereonite 7 was clearly visible. Pereopods 1–6 were bigger and more robust with a higher number of setae than in the M II stage (Fig. 9f–k). Pereopod 7 was visibly folded in the ventral position and the articles could be distinguished (Fig. 8b). Pleopod 1 was absent; pleopod 2 had one seta; pleopods 3 had seven setae, while pleopods 4–5 had ten setae (Fig. 9l–o).

Mean values of cephalothorax width, body length, and duration of the three stages are reported in Table 3.

**Table 2.** Summary of the main characteristics of the manca stages of *Porcellio siculoccidentalis*: ommatidia, antenna, pereopod 7, and pleopods exopodite.

**Manca Stage**	**No. of ommatidia**	**Antenna: flagellum**	**Pereopod 7**	**Pleopod 2–5 exopodite**
M I	4	Proximal article longer than the distal	absent	Distal margin with one seta
M II	5	Distal article longer than the proximal	folded ventrally;articles not distinguishable	Distal margin with 4–7 setae
M III	7	Distal article longer than the proximal	folded ventrally;articles distinguishable	Distal margin with 7–10 setae

**Table 3.** Mean values (±DS) of cephalothorax width, body length, and duration of each manca stage of *Porcellio siculoccidentalis*.

**Manca stage**	**Cephalothorax width (mm)**	**Body length (mm)**	**Duration**
M I	0.48 ± 0.018	1.788 ± 0.053	62 ± 8.49 min
M II	0.62 ± 0.019	2.023 ± 0.098	10 ± 0.94 days
M III	0.72 ± 0.04	2.465 ± 0.149	11.7 ± 1.45 days

## Discussion and conclusions

Recently, many papers have described different aspects of the reproductive biology of terrestrial isopods. They cover some topics relating to the female reproductive system (Warburg 2011a), gestation (ovaries, oocytes), incubation, and embryogenesis taking place in the marsupium (Milatovic et al. 2010; Appel et al. 2011). Other recent papers have reviewed some aspects of post-parturial events concerning breeding periods, strategies and patterns, parturition, and size and number of broods (Warburg 2011b). Some other related ecological subjects concerning the adults have also been recently reviewed (Kight 2008; Hornung 2011). Despite this wealth of newly published data, there are few descriptions of the postmarsupial manca stages of terrestrial isopods; these are very important steps that bring a species to the juvenile stages and then to the adult stage. The present study provides a complete description of the postmarsupial manca stages.

In this study, fecundity and fertility were evaluated as the number of eggs and embryos, respectively, inside the marsupium of the ovigerous females. Both parameters were positively correlated with the size of the females. Comparable data were previously reported for *Armadillo officinalis* Duméril, 1816, from Libya (AlJetlawi and Nair 1994). Data from females with similar body lengths showed higher fecundity values than fertility values, indicating that not all of the eggs developed into embryos.

Data regarding the number of manca released were similar to those that have been reported for *Armadillo officinalis* (AlJetlawi and Nair 1994) from Libya, and for some neotropical species of Oniscidea, including *Atlantoscia* *floridana* (Van Name, 1940), *Benthana cairensis* Sokolowicz, Araujo & Boelter, 2008 (Sokolowicz et al. 2008), *Balloniscus glaber* (Araujo and Zardo, 1996), and *Balloniscus sellowii* (Brandt, 1833) (Quadros et al. 2008).

**Figure 10.**
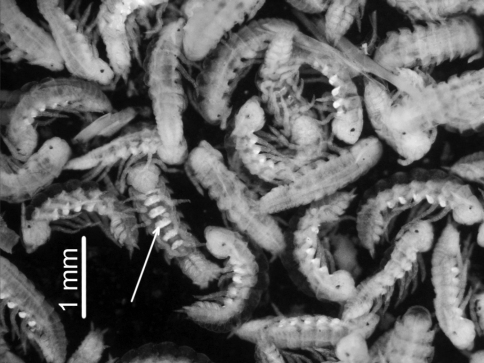
*Porcellio siculoccidentalis* Viglianisi et al., 1992. Specimens of manca stage M I, after the release from the brood pouch (arrow indicate calcium deposits).

In the present study, we observed that after release from the marsupium, the stage M I mancas were very thin and fragile and it was possible to observe sternal calcium deposits (Fig. 10) that mark the premolt stage (Zidar et al. 1998). This suggests that the M I mancas leave the marsupium in a premolt stage. For different terrestrial isopod species, Heeley (1941, 1942) first defined this stage as being characterized by incomplete segmentation, and a thin body with no evident pigmentation, characteristics that were also found in the manca I stage of *Porcellio siculoccidentalis*. This stage was also reported for *Atlantoscia floridana* (Araujo et al. 2004) and *Benthana cairensis* (Sokolowicz et al. 2008). The authors suggested that stage M I represents the last marsupial stage, when the animals did not molt or eat. In *Porcellio siculoccidentalis*, the first postmarsupial manca stage had a duration of only one hour, which is the shortest among the postmarsupial mancas previously described in the literature. For *Benthana cairensis*, the M I stage is 4-hours long, and the duration in other species varies from 12 hours for *Atlantoscia floridana* (Araujo et al. 2004), to 19 hours for *Porcellio dilatatus* Brandt, 1833 (Brum and Araujo 2007), to 48 hours for *Hemilepistus reaumurii* (Milne-Edwards, 1840) (Kacem-Lachkar 1997).

One observation from this study that, to our knowledge, has never been previously reported regards the mancas at the moment of the release from the marsupium. We observed that the newly released specimens at stage M I stayed under or near the female without moving until the first molt was completed. Only after molting (at stage M II) mancas started to move, exploring the environment and searching for food. In *Porcellio siculoccidentalis*, the mancas ate their exuviae after the first molting, as has been reported for other species (Helley 1941, Araujo et al. 2004, Brum and Araujo 2007, Sokolowicz et al. 2008).

One remarkable characteristic of stage M I regards the antennal flagellum. In this stage, the proximal article was longer than the distal; however, in the following manca stages, the proportions changed, and the distal article was longer than the proximal. This change is common during the developmental stages in different species belonging to various families of terrestrial isopods (Verhoeff 1920, Heeley 1941, Araujo et al. 2004, Brum and Araujo 2007). In *Porcellio siculoccidentalis*, the proportion changed again in the adults, where the distal article was shorter than the proximal (Fig. 5d).

During stage M II, the mancas were observed to feed normally, showing stronger and robust exoskeleton and pereopods; moreover, the seventh pereonite and pereopods developed, although the articles could not yet be distinguished. The premolt stage can be detected observing the calcium deposits in the pereon sternites (Zidar et al. 1998).

Stage M III was similar to stage M II with only a few differences. The seventh pereonite was larger than in M II. Additionally, the seventh pereopods were present in M III and showed all articles, although they were in a non-functional condition and remained folded ventrally under the body.

As previously mentioned, there are few papers in the literature that describe the manca stages of terrestrial isopods. However, the work already published clearly indicates that the major differences among the species relate to modifications of the appendages of the cephalothorax, pereon, and pleon (Verhoeff 1920, Haddad 1982, Araujo et al. 2004, Brum and Araujo 2007, Sokolowicz et al. 2008).

Here, three postmarsupial larval stages are described for *Porcellio siculoccidentalis*. After the third molt, the animals pass to the juvenile stages. The seventh pereopods become perfectly functional and the differentiation of the first pleopods begins. Lastly, the beginning of sexual differentiation can be observed.

Finally, in this study on *Porcellio siculoccidentalis*, we found no differences between the populations from Mount San Giuliano and Mount Inici. Specimens of both populations showed identical morphology of the appendages, and similar values were obtained regarding size, survival rate, number of manca released, fecundity, and fertility. No other differences were found on the reproductive biology of the two populations.

Comparative data obtained from the infected specimens from Mount San Giuliano will provide new information about any modifications caused by the presence of nematodes and/or Iridovirus infection. This will be a future task and a natural continuation of the present work.
